# Comparison of procedural efficacy, balloon nadir temperature, and incidence of phrenic nerve palsy between two cryoballoon technologies for pulmonary vein isolation: A systematic review and meta‐analysis

**DOI:** 10.1111/jce.15182

**Published:** 2021-07-26

**Authors:** Amira Assaf, Rohit Bhagwandien, Tamas Szili‐Torok, Sing‐Chien Yap

**Affiliations:** ^1^ Department of Cardiology, Erasmus MC University Medical Center Rotterdam Rotterdam The Netherlands

**Keywords:** atrial fibrillation, catheter ablation, cryoablation, cryoballoon, pulmonary vein isolation

## Abstract

**Introduction:**

In May 2020, a novel cryoballoon system (POLARx; Boston Scientific) became available for catheter ablation of atrial fibrillation (AF). The design of the cryoballoon is comparable to the Arctic Front Advance Pro (AFA‐Pro; Medtronic), but it is more compliant during freezing. We compared the procedural efficacy, biophysical parameters, and risk of phrenic nerve palsy (PNP) between the two cryoballoons.

**Methods:**

Embase, MEDLINE, Web of Science, Cochrane, and Google Scholar databases were searched until June 1, 2021 for relevant studies comparing POLARx versus AFA‐Pro in patients undergoing pulmonary vein isolation (PVI) for AF.

**Results:**

A total of four studies, involving 310 patients were included. There was no difference between the two groups for outcomes regarding procedural efficacy: acute PVI (odds ratio [OR]: 0.43; 95% confidence interval [CI]: 0.06 to 3.03; *p* = .40), procedure time (mean difference [MD]: 8.15 min; 95% CI: −8.09 to 24.39; *p* = .33), fluoroscopy time (MD: 1.32 min; 95% CI: −1.61 to 4.25; *p* = .38) and ablation time (MD: 1.00 min; 95% CI: −0.20 to 2.20; *p* = .10). The balloon nadir temperature was lower for all individual pulmonary veins (PV) in POLARx compared with AFA‐Pro (MD: −9.74°C, −9.98°C, −6.72°C, −7.76°C, for left superior PV, left inferior PV, right superior PV, and right inferior PV, respectively; all *p* < .001). The incidence of PNP was similar between groups (OR: 0.79; 95% CI: 0.22 to 2.85; *p* = .72).

**Conclusion:**

In AF patients undergoing PVI, POLARx and AFA‐Pro had a similar procedural efficacy. Balloon nadir temperatures were lower with POLARx, however, the incidence of PNP was similar.

**Abbreviations**
AF: atrial fibrillation
AFA‐Pro: Arctic Front Advance Pro
CI: confidence interval
LIPV: left inferior pulmonary vein
LSPV: left superior pulmonary vein
MD: mean difference
OR: odds ratio
PNP: phrenic nerve palsy
PV: pulmonary vein
PVI: pulmonary vein isolation
RIPV: right inferior pulmonary vein
RSPV: right superior pulmonary vein

## 1 INTRODUCTION

Pulmonary vein isolation (PVI) remains the cornerstone of catheter ablation of atrial fibrillation (AF).^1^ Among the different available single‐shot devices, cryoballoon has demonstrated to be as effective and safe as radiofrequency ablation for achieving PVI.^2^, ^3^, ^4^, ^5^, ^6^, ^7^, ^8^ Advantages of the cryoballoon in comparison to radiofrequency ablation is the shorter procedure duration and lower interoperator variability in outcomes.^2^, ^3^, ^4^, ^5^, ^6^, ^7^, ^8^ In May 2020, a novel cryoballoon was introduced, the POLARx cryoablation system (Boston Scientific). One of the unique features of this cryoballoon is that it maintains a uniform pressure and size during inflation and cryoablation. Several centers have published their initial clinical experience with this POLARx cryoablation system and have compared it with the fourth‐generation Arctic Front Advance Pro (AFA‐Pro) cryoballoon (Medtronic).^9^, ^10^, ^11^, ^12^ Most of these studies are limited by their small sample size, observational design, and single‐center design. A meta‐analysis may overcome part of these limitations and may provide more robust data. These data are relevant for operators interested in the performance of the novel POLARx cryoballoon in clinical practice.

### 1.1 Aim of the study

The aim of this comprehensive meta‐analysis was to compare the differences in procedural efficacy, balloon nadir temperature, and incidence of phrenic nerve palsy (PNP) between POLARx and AFA‐Pro in patients with AF undergoing PVI.

## 2 METHODS

### 2.1 Search strategy and study selection

This meta‐analysis was performed in accordance with the Preferred Reporting Items for Systematic Reviews and Meta‐Analysis literature search extension (PRISMA‐S) and Meta‐analysis Of Observational Studies in Epidemiology (MOOSE) checklists (Appendix SA).^13^, ^14^ The librarian‐mediated systematic search strategy of our center was previously described.^15^ The following electronic databases were searched on June 1, 2021: EMBASE (Ovid), MEDLINE (Ovid), Web of Science Core Collection (Web of Knowledge), Cochrane Central Register of Controlled Trials (Wiley), and Google Scholar. The search involved the following keywords: (“polarx” OR (“cryoablation” or “cryoballoon”) OR (“fourth‐generation” or “4^th^‐generation” or “4^th^‐CB” or “CB4” or “CBG4” or “arctic front” or “AFA‐Pro”)) AND (“pulmonary vein isolation” or “PVAI” or “PVI”). The complete search strategy per database is reported as Supporting Information (Appendix SB). We also searched ClinicalTrials.gov to identify ongoing trials. The search was limited to the English language and adult (18 years or older) human participants. All searches were limited to publications from 2019 to 2021 given that the POLARx cryoballoon was only commercially available in May 2020. Reference lists of included studies were manually screened to identify additional studies.

### 2.2 Eligibility criteria

The studies included fulfilled the following criteria: (1) patients with paroxysmal and/or persistent AF undergoing PVI with a cryoballoon; (2) comparison of POLARx cryoballoon with AFA‐Pro cryoballoon; and (3) reported outcome data including but not limited to acute PVI success, procedure time, fluoroscopy time, ablation time, balloon nadir temperature for each pulmonary vein (PV), and PNP. The following exclusion criteria were used: conference abstracts, case reports, review articles, editorials, and letters to the editor. Two reviewers screened articles using EndNote for inclusion independently, retrieved potentially relevant articles, and determined their eligibility.^16^ Disagreements were resolved through consensus, and consultation of a third reviewer if necessary.

### 2.3 Data abstraction, data extraction, and quality assessment

The following baseline patient characteristics were extracted from each included study: age, sex, type of AF, hypertension, diabetes, coronary artery disease, and left atrial size. Extracted outcome data for procedural efficacy included acute PVI success, procedure time, fluoroscopy time, and ablation time. The following biophysical parameter was extracted per individual pulmonary vein: balloon nadir temperature. Finally, the occurrence of PNP was extracted from each included study. No authors were contacted as all relevant variables could be extracted from the published article. The quality of studies used in the analysis was assessed using the Newcastle Ottawa scale. Two reviewers independently performed data extraction and assessed study quality. Disagreements were resolved through consensus, and consultation of a third reviewer if necessary.

### 2.4 Statistical analysis

For continuous outcome variables, the pooled mean difference (MD) and the corresponding 95% confidence intervals (CI) were estimated using the inverse‐variance method. If a study provided medians and interquartile ranges or ranges, we estimated the means and SDs using Wan et al.'s^17^ method for the purpose of this meta‐analysis. For categorical outcome variables, the pooled odds ratio (OR) and corresponding 95% CI were estimated using Mantel–Haenszel random‐effects model.^18^ A random‐effects model was chosen a priori on the basis of the anticipated heterogeneity in baseline characteristics. Two‐sided *p* < .05 was considered statistically significant. The presence of statistical heterogeneity was evaluated by Cochran's Q test *I* ^2^ statistic. Statistical analysis was performed using Review Manager (RevMan, version 5.4.1., the Nordic Cochrane Centre, The Cochrane Collaboration, 2020).

## 3 RESULTS

### 3.1 Search results and baseline characteristics

**Figure 1:**
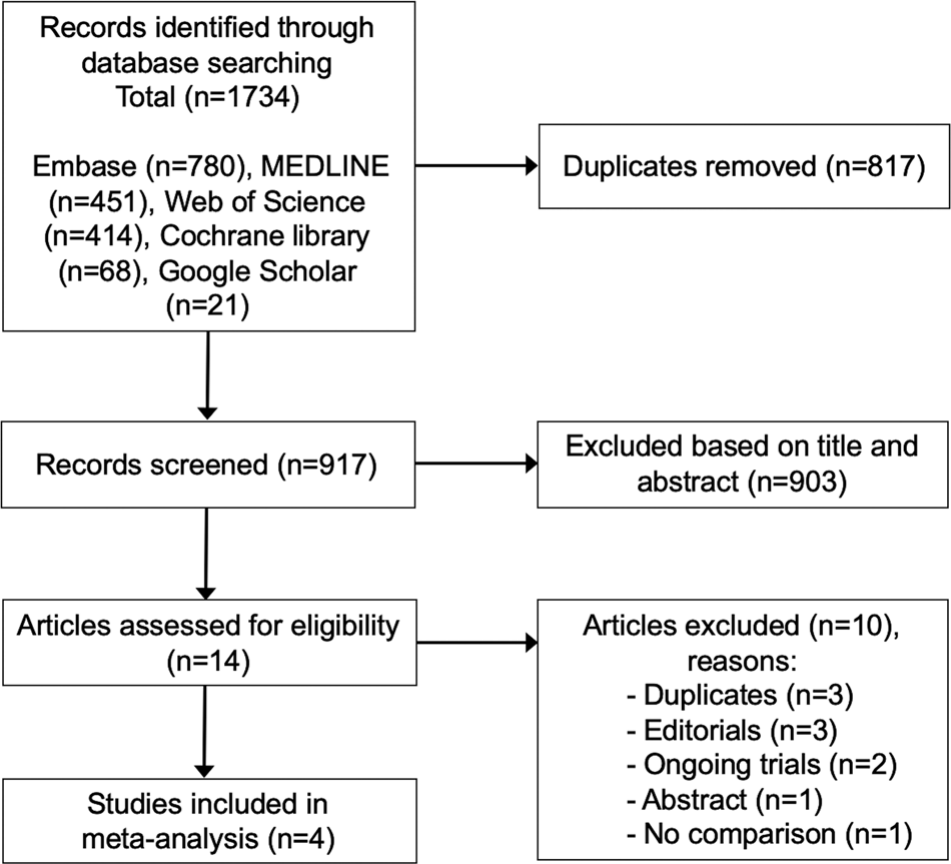
PRISMA (Preferred Reporting Items for Systematic Reviews and Meta‐Analysis) flow chart for the selection of studies included in this meta‐analysis

Among 917 unique citations, 14 citations were retrieved for full‐text review. Following the review, a total of four studies met inclusion criteria (Figure 1).^9^, ^10^, ^11^, ^12^ All four included studies were observational in design and found to be of good quality based on the Newcastle Ottawa scale (Table S1).

In total, 310 patients were included in the analysis of whom 142 and 168 patients underwent ablation with the POLARx and AFA‐Pro system, respectively. The characteristics of the included studies are summarized in Table 1. Baseline patient characteristics among the included studies are shown in Table 2. The mean or median age of the patients ranged from 61 to 69 years and the proportion of males ranged from 52% to 84%. The proportion of patients with paroxysmal AF ranged from 36% to 95%.

**Table 1:** Studies included in the meta‐analysis

Study	Year of publication	Center	Design	Freezing protocol	Bonus freeze	Number of patients in POLARx group	Number of patients in AFA‐Pro group	Details of POLARx group	Details of AFA‐Pro group
Creta et al.^9^	2021	Barts Heart Center (UK)	Single‐center, non‐RCT	≥180 s/PV	No	40	40	Consecutive cohort	Consecutive cohort
Kochi et al.^10^	2021	Centro Cardiologico Monzino IRCCS (Italy)	Single‐center, non‐RCT	≥180 s/PV	No	20	50	Consecutive cohort from August to October 2020	Consecutive cohort from October 2018 to February 2019
Tilz et al.^11^	2021	University Heart Center Lübeck (Germany)	Single‐center, non‐RCT	180 s/PV if TTI <60 s	Only if TTI >60 s	25	25	Consecutive cohort from August to October 2020	Consecutive cohort from May to July 2020
Yap et al.^12^	2021	Erasmus MC (NL), Clinical Hospital Center Split (Croatia), Städtisches Klinikum Karlsruhe (Germany)	Multi‐center, non‐RCT	180 s/PV if TTI <60 s, otherwise 240 s	No	57	53	Consecutive cohort from May to October 2020	Consecutive cohort from May to October 2020

Abbreviations: PV, pulmonary vein; RCT, randomized controlled trial; TTI, time‐to‐isolation.

**Table 2:** Baseline characteristics of studies included in the meta‐analysis

	Age (years)		Male sex (%)		Paroxysmal AF (%)		Hypertension (%)		Diabetes (%)		Left atrial size	
Study	P	A	P	A	P	A	P	A	P	A	P	A
Creta et al.^9^	63	65	65	60	70	48	43	35	3	3	40 mm	38 mm
Kochi et al.^10^	63	61	60	84	95	94	60	30	5	6	36 ml/m^2^	33 ml/m^2^
Tilz et al.^11^	68	69	52	68	48	36	80	72	12	12	25 ml/m^2^	29 ml/m^2^
Yap et al.^12^	61	64	58	68	75	76	32	59	5	6	41 mm	41 mm

Abbreviations: A, Arctic Front Advance Pro; AF, atrial fibrillation; P, POLARx.

### 3.2 Pooled analysis

**Figure 2:**
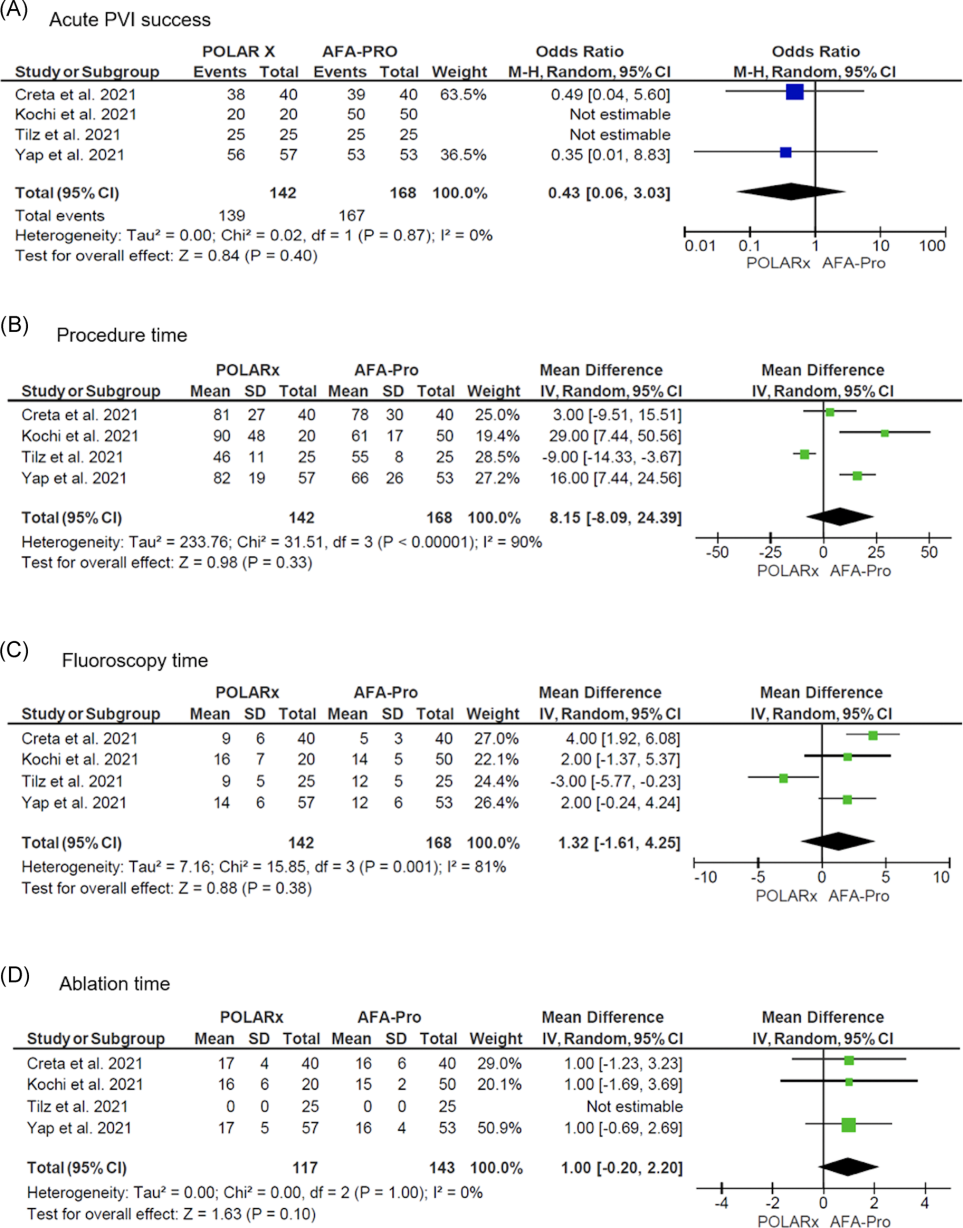
Forest plots of the pooled analysis demonstrating the effect of POLARx versus AFA‐Pro on procedural efficacy in patients with atrial fibrillation (AF). For acute pulmonary vein isolation (PVI) success, events and weighted odds ratios are presented. For continuous outcomes, mean, SD, and mean differences are presented. The horizontal line is the 95% confidence interval (CI). The diamond shape is the estimate and the CI of the estimate. (A) acute PVI success; (B) procedure time; (C) fluoroscopy time; (D) ablation time

There was no difference in achieving successful acute PVI between the two groups (OR: 0.43; 95% CI: 0.06 to 3.03; *p* = .40). There was also no statistically significant difference between the two groups in the outcome of procedure time (MD: 8.15 min; 95% CI: −8.09 to 24.39; *p* = .33), fluoroscopy time (MD: 1.32 min; 95% CI: −1.61 to 4.25; *p* = .38), and ablation time (MD: 1.00 min; 95% CI: −0.20 to 2.20; *p *= .10). Forest plots regarding procedural efficacy are shown in Figure 2.

**Figure 3:**
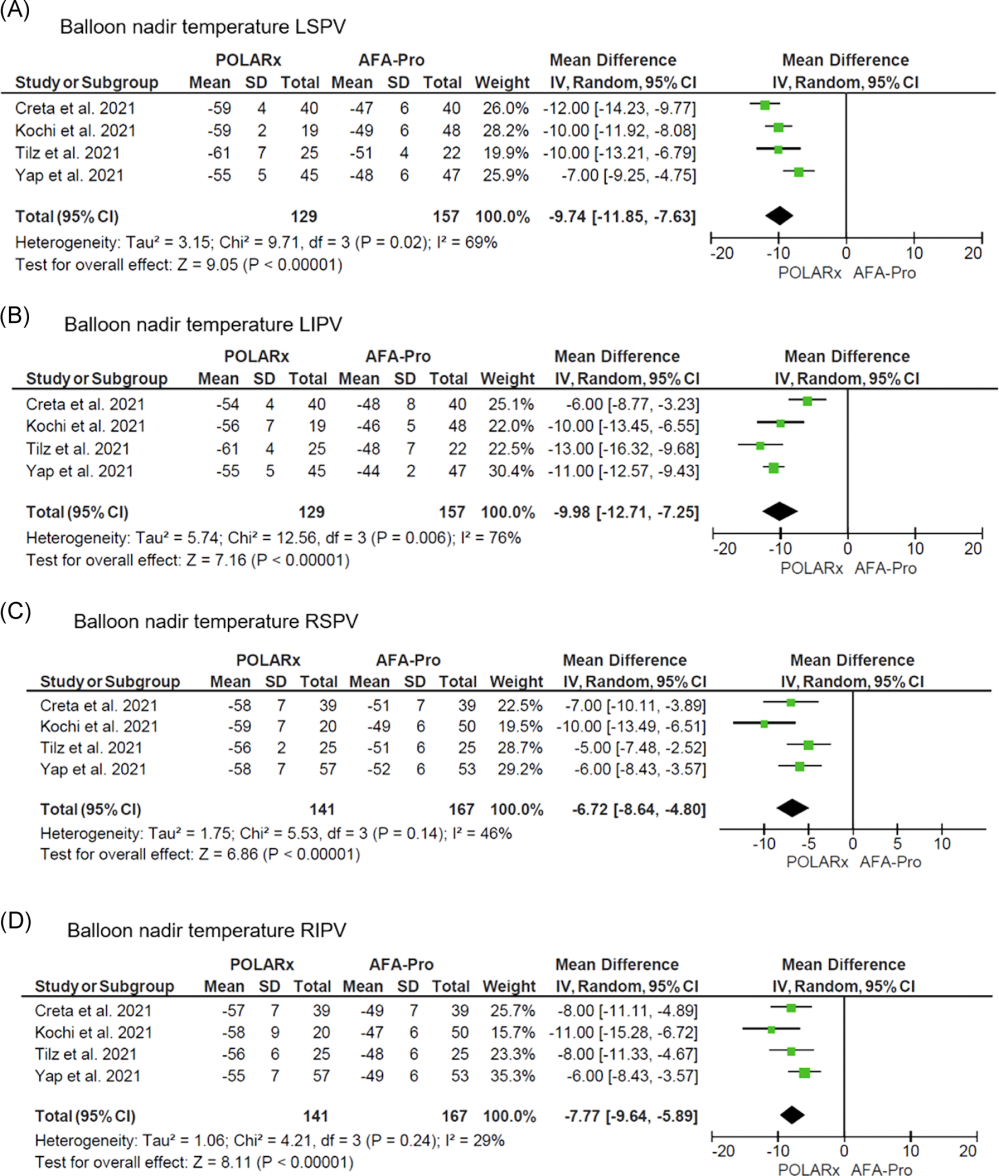
Forest plots of the pooled analysis demonstrating the effect of POLARx versus AFA‐Pro on balloon nadir temperature in patients with AF. The data are presented as mean, standard deviation, and mean difference. The horizontal line is the 95% CI. The diamond shape is the estimate and the confidence interval of the estimate. AF, atrial fibrillation; CI, confidence interval; LIPV, left inferior pulmonary vein; LSPV, left superior pulmonary vein; RIPV, right inferior pulmonary vein, RSPV, right superior pulmonary vein

**Figure 4:**
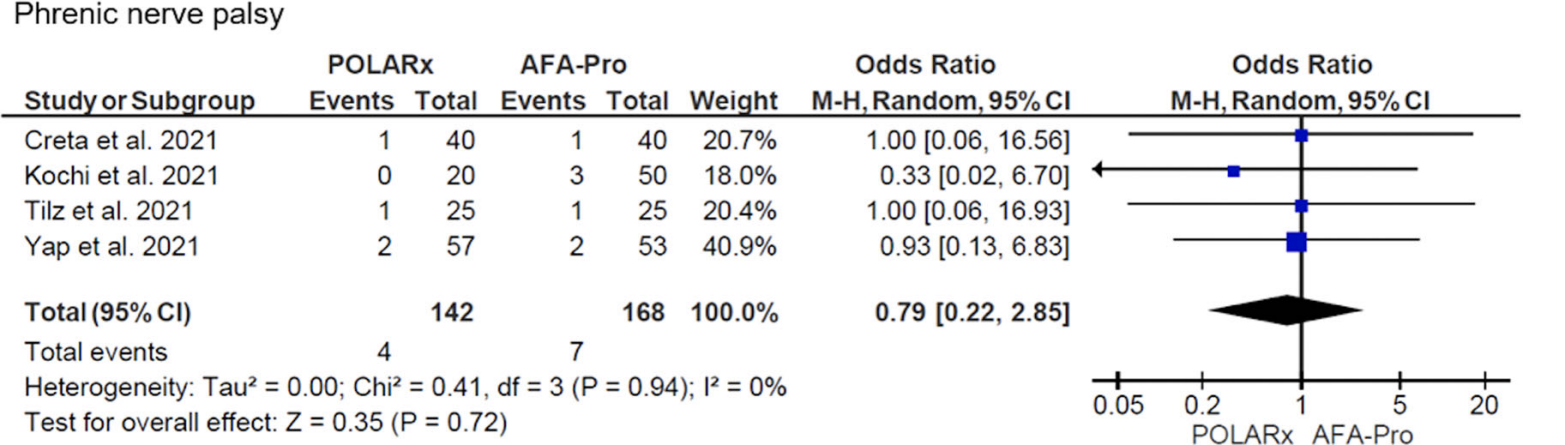
Forest plot of the pooled analysis demonstrating the effect of POLARx versus AFA‐Pro on phrenic nerve palsy (PNP) in patients with atrial fibrillation (AF). The data are presented as events and weighted odds ratios. The horizontal line is the 95% confidence interval (CI). The diamond shape is the estimate and the confidence interval of the estimate

The pooled balloon nadir temperature was lower with POLARx in comparison to AFA‐Pro for all individual PVs (Figure 3): left superior PV (LSPV) (MD: −9.74°C; 95% CI: −11.85 to −7.63; *p* < .001); left inferior PV (LIPV) (MD: −9.98°C; 95% CI: −12.71 to −7.25; *p *< .001); right superior PV (RSPV) (MD: −6.72°C; 95% CI: −8.64 to −4.80; *p* < .001); and right inferior PV (RIPV) (MD: −7.77°C; 95% CI: −9.64 to −5.89; *p* < .001). There was no difference in the incidence of PNP between the two modalities (OR: 0.79; 95% CI: 0.22 to 2.85; *p* = .72) (Figure 4).

### 3.3 Sensitivity analysis

There was significant statistical heterogeneity (*I* ^2^ ≥ 50%) for the outcomes of procedure time (90%), fluoroscopy times (81%), balloon nadir temperature in the LSPV (69%), and balloon nadir temperature in the LIPV (76%). For the outcome procedure time, the between‐study heterogeneity remained high (*I* ^2^ ≥ 50%) with the sequential exclusion of studies. Heterogeneity for the outcome of fluoroscopy time was driven primarily by the study of Tilz et al.^11^ Sensitivity analysis demonstrated a higher fluoroscopy time for POLARx after the exclusion of the study of Tilz et al. (MD: 2.89 min; 95% CI: 1.50 to 4.28; *p* < .001; *I* ^2^: 0%) (Figure S1). Heterogeneity for the outcome of balloon nadir temperature in the LSPV and LIPV was driven primarily by the study of Yap et al. and Creta et al., respectively. Sensitivity analysis demonstrated robust results with marginal change in the pooled MD balloon nadir temperature in the LSPV and LIPV (Figure S1). Balloon nadir temperatures remained lower with POLARx in comparison to AFA‐Pro. No funnel plots were constructed to examine publication bias due to the low number of included studies (<10). The power of the test would be too low to distinguish chance from real asymmetry.

## 4 DISCUSSION

This meta‐analysis demonstrates that patients with symptomatic AF undergoing cryoballoon ablation have a similar acute procedural efficacy with either the POLARx or AFA‐Pro system. Despite a lower balloon nadir temperature with POLARx, the incidence of PNP is similar to AFA‐Pro.

Cryoballoon ablation has been shown to be effective for PVI in patients with AF and to be noninferior to radiofrequency ablation.^2^, ^3^, ^4^, ^5^, ^6^, ^7^, ^8^ The AFA‐Pro is the latest generation cryoballoon from Medtronic and is currently the most widely used cryoballoon. In May 2020, a novel cryoballoon system, POLARx (Boston Scientific), became commercially available. Similar to AFA‐Pro, it consists of a double‐layer balloon of 28 mm and has eight refrigerant injection ports resulting in cooling of the entire distal half of its surface. The main difference between the two cryoballoon technologies is the constant pressure inside the POLARx balloon.

The more compliant balloon of POLARx may have an ambiguous effect on procedural efficacy because operators may have to adjust their procedural workflow. This is reflected in the high between‐study heterogeneity for procedure time. Tilz et al.^11^ demonstrated a trend toward a shorter procedure time with POLARx, potentially secondary to a combination of stable balloon size during inflation and ablation, foot pedal, slider switch, and POLARSHEATH according to the authors. In contrast, Yap et al. and Kochi et al. showed a longer procedure time with the POLARx system.^10^, ^12^ A learning curve effect was demonstrated by Yap et al. as the procedure times between both platforms were similar in the second half of the study cohort.^12^ The current meta‐analysis did not demonstrate a significant difference in procedural efficacy between POLARx and AFA‐Pro in terms of acute PVI success, procedure time, fluoroscopy time, and ablation time. Considering that all included studies reported their initial experience with the POLARx cryoballoon (<50 cases per center), it seems that the use of this novel cryoballoon is relatively straightforward in centers with experienced cryoballoon users.

Despite similarities in balloon shape and thermal energy source, the balloon nadir temperature with POLARx was significantly lower than AFA‐Pro. There was little between‐study heterogeneity for the difference in balloon nadir temperature in the right‐sided PVs. There was significant between‐study heterogeneity for the difference in balloon nadir temperature in the left‐sided PVs. However, sensitivity analysis demonstrated robust results. The MD between POLARx and AFA‐Pro in balloon nadir temperatures ranged from −7°C to −10°C depending on PV location. Balloon nadir temperature is only a surrogate marker for the target atrial tissue temperature. Besides balloon nadir temperature, other factors affecting target atrial tissue temperature are balloon–tissue contact area, balloon‐to‐PV size ratio, and ipsilateral PV blood flow. A previous study has shown that the median balloon temperature at time‐to‐isolation was lower with POLARx in comparison to AFA‐Pro (−46°C vs. −37°C; *p *< .001); a difference of approximately 10°C.^12^ This could imply that to achieve the desired biological effect (i.e., PV isolation) a lower measured balloon temperature is needed with POLARx in comparison to AFA‐Pro. This is important for clinicians as biophysical parameters associated with durable PVI established with AFA‐Pro may potentially not be applicable for POLARx.^19^ More research on the relationship between biophysical parameters of POLARx and durable PVI is necessary to identify target values that are associated with a high likelihood of durable PVI.

PNP is the most frequently observed complication during cryoballoon ablation. The POLARx system has incorporated a diaphragmatic movement sensor to follow the diaphragmatic movement during cryoablation. With AFA‐Pro, manual palpation of diaphragmatic movement or compound motor action potential monitoring is used. In the current meta‐analysis there was no difference in the incidence of PNP between POLARx and AFA‐Pro, despite lower balloon nadir temperatures in the right‐sided PVs with POLARx. Again, as mentioned previously, we do not know if there is a difference in target atrial tissue temperature between both platforms. Most patients recover from PNP during long‐term follow‐up using the fourth‐generation AFA‐Pro.^20^ We expect that PNP recovery will also occur in the majority of patients using POLARx, however, currently there is limited published data on the long‐term outcome of acute PNP with this novel system.^21^ The study of Anic et al. presented 1‐year follow‐up data of 24 patients undergoing cryoablation with POLARx. In this study, only one patient experienced transient PNP that recovered intraprocedurally.

## 5 STUDY LIMITATIONS

All studies included in this meta‐analysis were observational studies, but they were of good quality based on the Newcastle Ottawa scale. Ideally, the results of our meta‐analysis should be confirmed in randomized controlled trials. Currently, there are two ongoing randomized controlled trials comparing POLARx and AFA‐Pro for the treatment of paroxysmal AF (NCT04704986, ACTRN12621000003875); however, both trials are not recruiting patients according to the latest update (June 1, 2021). For some outcome parameters, there was significant heterogeneity between studies, but to account for this we used a random‐effects model a priori. We report only on the acute outcome; thus, we do not have data on long‐term outcome such as persistent PNP and freedom from atrial arrhythmia. This limitation is inherent to the recent introduction of the POLARx cryoballoon. Furthermore, it was not possible to provide a forest plot for time‐to‐isolation as most studies did not provide data on how often time‐to‐isolation could be recorded. Finally, considering the selection of centers for limited market release of the POLARx cryoballoon who published their initial experience, the results of this meta‐analysis should be viewed with caution as it may not be generalizable to other centers.

## 6 CONCLUSION

The novel POLARx cryoballoon is comparable to AFA‐Pro with regard to procedural efficacy in terms of acute PVI, procedural time, fluoroscopy time, and ablation time. Although the balloon nadir temperatures were significantly lower with POLARx, the risk of PNP was comparable to AFA‐Pro.

## Supporting information

Sensitivity analysis. Forest plots of the pooled analysis demonstrating the effect of POLARx versus AFA‐Pro. The data are presented as mean, standard deviation and mean difference. The horizontal line is the 95% CI. The diamond shape is the estimate and the confidence interval of the estimate. Abbreviations: LIPV, left inferior pulmonary vein; LSPV, left superior pulmonary vein.

Supporting information.

Supporting information.

Supporting information.

## Data Availability

The data that support the findings of this study are available from the corresponding author upon reasonable request.
