# Supplementary figures and images for: Comparison of procedural efficacy, balloon nadir temperature, and incidence of phrenic nerve palsy between two cryoballoon technologies for pulmonary vein isolation: A systematic review and meta‐analysis
Source: J Cardiovasc Electrophysiol. 2021 Jul 26;32(9):2424–31. doi: 10.1111/jce.15182 (PMC9292548; doi:10.1111/jce.15182)

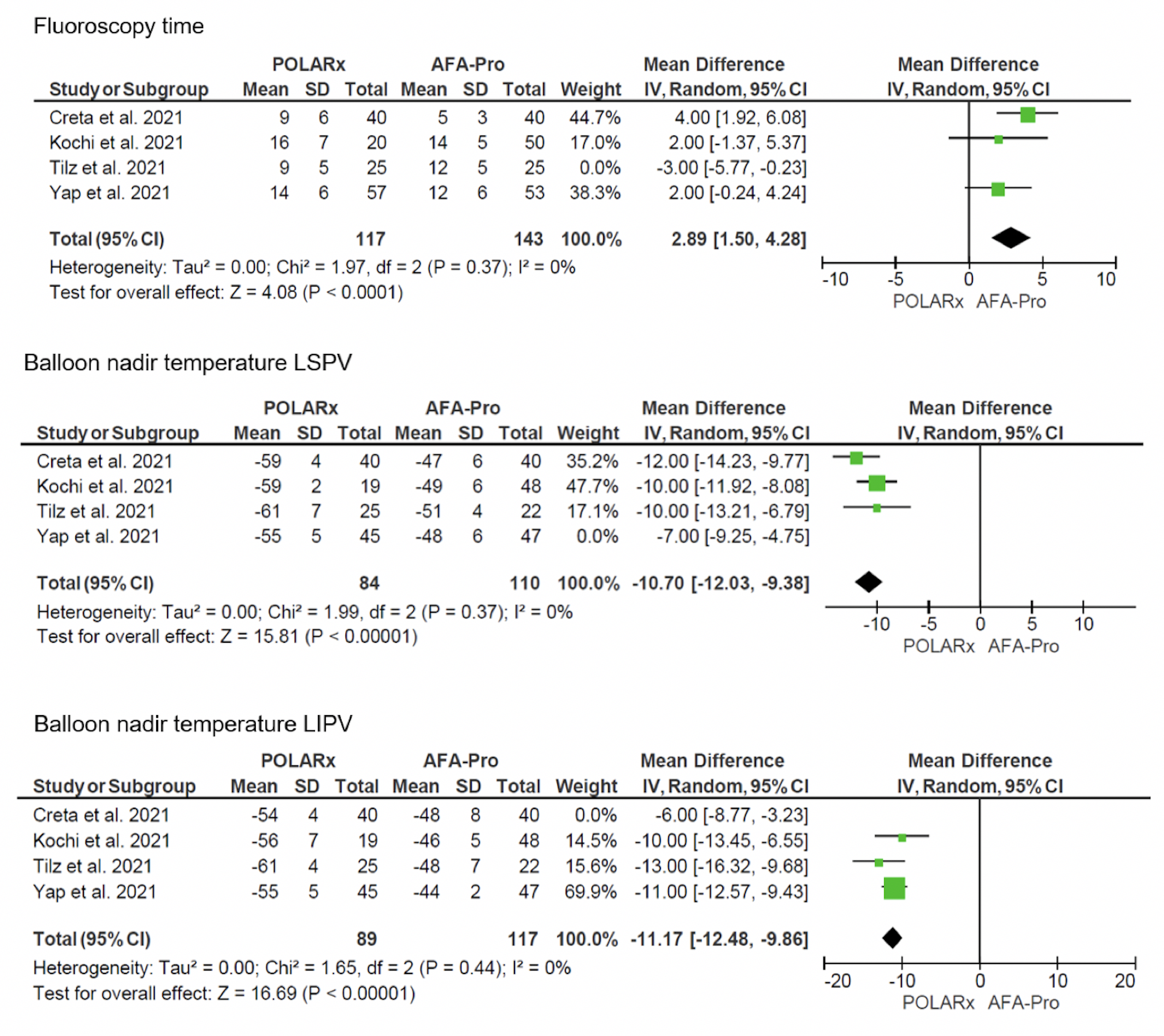

Supplement: Supplementary file 1 — Sensitivity analysis. Forest plots of the pooled analysis demonstrating the effect of POLARx versus AFA‐Pro. The data are presented as mean, standard deviation and mean difference. The horizontal line is the 95% CI. The diamond shape is the estimate and the confidence interval of the estimate. Abbreviations: LIPV, left inferior pulmonary vein; LSPV, left superior pulmonary vein. [file JCE-32-2424-s002.tiff]
