# Supplementary material for: Comparison of procedural efficacy, balloon nadir temperature, and incidence of phrenic nerve palsy between two cryoballoon technologies for pulmonary vein isolation: A systematic review and meta‐analysis
Source: J Cardiovasc Electrophysiol. 2021 Jul 26;32(9):2424–31. doi: 10.1111/jce.15182 (PMC9292548; doi:10.1111/jce.15182)
Supplement: Supplementary file 2 — Supporting information. [file JCE-32-2424-s003.docx]

**Supplemental table 1**. Newcastle-Ottawa quality assessment for observational studies

| **Study** | **Selection** | **Comparability** | **Outcome** | **Score** | **Study quality** |
| --- | --- | --- | --- | --- | --- |
| Creta et al.^9^ | 🟋🟋🟋🟋 | 🟋 | 🟋🟋🟋 | 8 | Good |
| Kochi et al.^10^ | 🟋🟋🟋🟋 | 🟋 | 🟋🟋🟋 | 8 | Good |
| Tilz et al.^11^ | 🟋🟋🟋🟋 | 🟋 | 🟋🟋🟋 | 8 | Good |
| Yap et al.^12^ | 🟋🟋🟋🟋 | 🟋 | 🟋🟋🟋 | 8 | Good |
